# Electrochemical and Computational Approaches of Polymer Coating on Carbon Steel X52 in Different Soil Extracts

**DOI:** 10.3390/polym14163288

**Published:** 2022-08-12

**Authors:** Hana Ferkous, Amel Delimi, Abdesalem Kahlouche, Chérifa Boulechfar, Souad Djellali, Amina Belakhdar, Krishna Kumar Yadav, Ismat H. Ali, Akil Ahmad, Hyun-Jo Ahn, Magda H. Abdellattif, Byong-Hun Jeon, Yacine Benguerba

**Affiliations:** 1 Laboratoire de Génie Mécanique et Matériaux, Faculté de Technologie, Université de 20 Août 1955 de Skikda, Skikda 21000, Algeria; 2 Département de Technologie, Université de 20 Août 1955 de Skikda, Skikda 21000, Algeria; 3 CRTI Research Centre in Industrial Technologies-CRTI, P.O. Box 64, Cheraga, Algiers 16014, Algeria; 4 Laboratoire de Physico-Chimie des Hauts Polymères (LPCHP), Faculty of Technology, University Ferhat Abbas Setif1, Setif 19000, Algeria; 5 Laboratoire Matériaux et Systèmes Electroniques, Université de Bordj Bou Arreridj, Bordj Bou Arreridj 34000, Algeria; 6 Faculty of Science and Technology, Madhyanchal Professional University, Ratibad, Bhopal 462044, India; 7 Department of Chemistry, College of Science, King Khalid University, P.O. Box 9004, Abha 61413, Saudi Arabia; 8 Chemistry Department, College of Science and Humanities, Prince Sattam Bin Abdulaziz University, Al-Kharj 11942, Saudi Arabia; 9 Department of Earth Resources and Environmental Engineering, Hanyang University, Seoul 04763, Korea; 10 Department of Chemistry, College of Science, Taif University, Al-Haweiah, Taif 21944, Saudi Arabia; 11 Laboratoire de Biopharmacie et Pharmacotechnie (LBPT), Ferhat Abbas Setif 1 University, Setif 19000, Algeria

**Keywords:** soil extract, steel, coated system, corrosion, electrochemical impedance spectroscopy, DFT

## Abstract

Using stationary electrochemical, polarization resistance, cathodic charging, transient electrochemical impedance spectroscopy, and theoretical and molecular mechanics studies, epoxy polymer-coated carbon steel specimens’ ability to protect metals from corrosion in various soil extracts was examined. According to the polarization resistance tests results, the polymer coating remained stable for 60 days in all three soil extracts, with a 90% efficiency for the steel coated in Soil Extract A, indicating that the sandy soil is less aggressive than the other two. The aggressiveness of clay soil was confirmed by the fact that a polymer-coated steel rod in the clay soil extract experienced a corrosion current density of 97 µA/cm^2^. In contrast, the same rod in sandy soil had a current density of 58 µA/cm^2^. The coating’s good adsorption contact with the metal surface was further guaranteed by molecular dynamics simulations, which provided atomic-level evidence of the epoxy molecule’s adsorption behavior (geometry) and adsorption energy on the carbon steel surface.

## 1. Introduction

Polymer or paint coatings have been used for decades to protect metals against corrosion, and this strategy is the most effective. It is best to use water-borne (WB) and solvent-borne (SB) paint coatings to protect metals against corrosion. An essential factor in paint coating protection is the inclusion of specific additives to the base film, such as anti-corrosive pigments and corrosion inhibitors, in the paint coating formulation process. Corrosion-inhibiting components in the paint may be separated into organic and inorganic substances.

Corrosion-inhibiting agents of the adsorption or oxidative type are more often utilized in WB than in SB [1,2,3,4]. An essential quality of any paint is its ability to adhere to various surfaces [5,6]. With the addition of a bifunctional chemical, the adherence of polymer films to metal may be enhanced. One functional half of a molecule interacts with the polymer or paint components (i.e., undergoing polymerization). One is also engaged in building a chemical connection simultaneously with the metal [5,7]. Polymers from thermoplastic are used in various coated systems, including powder coatings [7], automobile anti-corrosive paints, and clean room applications [8].

For example, polyethylene (homopolymers and copolymers ethylene-propylene), polyamide (PA66), polyester (PET, PBT), and polycarbonate (PC) have been extensively employed. Solvent-borne paints are most often utilized in alkyd resins [9]. Alkyd and epoxy resin’s corrosion resistance has been improved using conductive polymers such as polyaniline. The passivation of the metal’s surface is proposed as the mechanism for polyaniline’s protective action [10,11]. Field and laboratory testing by Iribarren et al. [12] investigated the corrosion of CS in coastal and urban settings. The availability of an equal period in the laboratory accelerated for field tests proved a satisfactory connection. Three thermoplastic systems (vinyl copolymer, a mix of two acrylic polymers, and the phenoxy resin) were chosen to test corrosion resistance and compare the results with an alkyd polymer modified with polyaniline as a conductive polymer, according to J.I. Iribarren and coworkers [13]. Developing corrosion-resistant materials is complicated because the polymer composition raises new challenges [14,15]. Molecular design components for excellent adhesion, good barrier qualities, and efficient pigment utilization in organic coatings are often at odds, as discussed earlier in this study. There is no theoretical foundation for resolving these competing issues. Corrosion-control product development requires an empirical balance of attributes to be made. Unsurprisingly, the composition specifics important to performance frequently remain confidential or only surface in the patent literature. Corrosion is a natural electrochemical phenomenon that damages materials over time and causes significant financial losses for various companies [16,17]. In 2016, NACE calculated the global cost of corrosion to be USD 2.5 trillion (3.4% of global output) [18,19]. For example, it has been predicted that using current corrosion prevention techniques can save 15% to 35% [20]. Figures like these show the importance of corrosion prevention measures in improving metallic structures’ service lives and lowering the need for repairs in industries [21].

The construction sites for most of the world’s gas pipelines are exposed to typical weather and topographical conditions. Conventional laying methods are still used, and even though the various construction processes have remained unchanged for many years, progressive improvements have been made in their implementation: trenching, pipe welding, track preparation, maintenance, etc. However, improvements have been made throughout their execution, such as better pipe coating operations, welding techniques, and weld inspection procedures (X-ray, ultrasound, etc.). Tube production techniques and steel with rated qualities have undergone extensive development to ensure high product quality and low failure rates.

One environmental factor that affects soil corrosion is the amount of water in the soil. The quantity of water in the soil affects the gas-to-liquid phase ratio. Because of the gas phase, O_2_ may be able to spread through the soil. Electrochemical reactions occur in the liquid phase at the steel-to-soil contact. Aside from being an electrolyte in the case of localized corrosion, it may also be used as a catalyst. Soil moisture content and corrosion rate have been the subject of several investigations [22,23,24]. CS corrodes the fastest, as much as 60% of its water-holding capacity [5]. The relationship between corrosion rate and soil moisture content was examined [25]. Depending on the saturation level, the research found that sand, silt, and clay had maximum corrosion rates of 50%, 70%, and 80%, respectively. Electrochemical and weight loss data were used to estimate typical study corrosion rates [26,27]. For this reason, metal surfaces in the soil come into direct contact with all three phases: solid soil particles, liquid groundwater, and gaseous air (air). Sometimes, the metal/soil contact area and the bulk soil may not be evenly saturated with water.

Additionally, the surface area in contact with water may be rather small in soil with a minimal volume. In any part of the world, aridity is never an issue. According to research [27,28], a CS-grade X52’s corrosion resistance in damp soil ranged from 10% to 45%, with X52’s corrosion rate peaking at 20% to 25%. It is passivated with a moisture level of 45%. We are interested in three parts of the GK140 pipeline in Ali MendjeliConstantine (A), Hamadi Krouma Skikda (B), and Bouzaroura Skikda (C). This conclusion is backed by the location in exceptionally hostile settings, leading to significant corrosion, pipe perforation, and serious safety and financial consequences.

## 2. Materials and Methods 

### 2.1. Materials

In this work, the steel specimens were cut from a X52 steel pipe line with the chemical composition (wt%): C (0.24%), Si (0.45%), Mn (1.4%), P (0.025%), Cu (0.16%), Cr (0.5%), S (0.015%), v (0.10%), (T0.04%), (Nb0.05%), and Fe balance.

### 2.2. Surface Coating

The samples were prepared as follows.

#### 2.2.1. Sanding

The air supplied by a compressor used for sanding must be free of all traces of oil and water. It must provide a continuous airflow of 7 bar. The use of good-quality sand and the origin and quality of the abrasives shall be set as a minimum as follows: river silica sand washed, dried with sharp edges, and of a size corresponding to the 20 to 40 mesh of the U.S. standards, whose granulometry allows obtaining a roughness of about 25 to 50 micro or iron shot. Before painting, the metallic substances were stripped by blasting with abrasives type AS 2.5, according to the standard ISO 8501-1, up to a degree of roughness (medium G) according to the standards NFEN-ISO 8503-2. The treated surfaces were cleaned immediately before applying the primer.

#### 2.2.2. Coating System

The paint system was deposited on the steel substrate and used for long-term immersion tests under different protection conditions, a commercial epoxy paint. The epoxy paint system is an epoxy coating that hardens at a temperature of 80 °C (Table S1).

**Figure 1:**
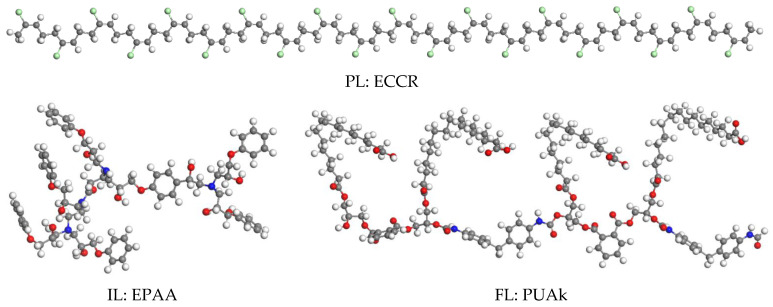
Optimized structures of the polymers constituting the three coating layers (PI, IL, FL).

Primer layer (PL): epoxy/chloric chlorinated rubber (ECCR) marine paint, applied as an undercoat (first layer) anticorrosion, rich in zinc powder (Figure 1). After preparing the steel surface (Sa 2.5 sandblasting), the PL is applied immediately by brush or roller with a thickness of 35 µm.

Intermediate layer (IL): The IL is a white epoxy/polyaminoamide (EPAA), reinforcing the system’s corrosion resistance. After the PL has dried in two 40 µm layers, they are applied by a roller or a pneumatic spray gun. Their primary role is to protect the PL against the access of water, oxygen, and ions. Hence, their name is synonymous with the sealing layer.

Final layer (FL): The FL consists of a two-component topcoat for the industrial epoxy system composed of polyurethane and alkyd (PUAk). After the IL coat has dried, it is applied in two 50 µm coats by brush, roller, or spray gun.

#### 2.2.3. Soil Sampling Procedure:

The pipe was placed at 1.60 m from the lower generatrix based on its diameter. The soil layer from the surface to the upper generatrix, covering the tube, is between 53 and 73 cm. Therefore, taking soil samples (Table 1) at a depth greater than or equal to 50 cm is recommended.

**Preparation** 

The solution we used for our experiments is an extract of soils taken from different sites: Ali Mendjelli (Constantine), Hamadi Krouma (Skikda), and Larbi ben M’hidi (Skikda).

Drying the soil sample in an oven at 105 °C for 2 h. Sieving of the soil to obtain a fineness suitable for uniform volumetric measurement. The volume of soil obtained is diluted in an equivalent volume of water (dissolution of salts). The mixture obtained is stirred for 1 h. The mixture was filtered using the gravitation method with filter papers or the vacuum method with a vacuum pump. The soil extract was prepared according to the procedure (Afnor standard A-05250P.278).

### 2.3. Surface Characterization

#### 2.3.1. Scanning Electron Microscopy 

SEM images were captured using a Zeiss ULTRA 55 electron microscope with a thermal field emission emitter and three detectors (ESB detector with filter grid, high-efficiency In-lens SE detector, Everhart–Thornley secondary electron detector).

#### 2.3.2. Ellipsometry

A Jobin Yvon Horiba Spectroscopic Ellipsometer equipped with DeltaPsi 2 data processing software was used to collect spectroscopic ellipsometry data in the visible range. The apparatus captured a spectrum with 0.05 eV (or 7.5 nm) intervals spanning from 2 to 4.5 eV (equivalent to 300 to 750 nm). The angle of incidence was set at 70°, and the compensator was adjusted to 45° for the measurements. We found that the film thicknesses and the two complicated refractive indices best represented a film-on-substrate model.

#### 2.3.3. Adhesion 

The performance of painted systems depends on the adhesion (See Supplementary Materials) between the substrate and the different layers of the paint system. The standard French ISO: 2409-1994 [29] gives all the details on the method known as the grid method. This destructive test makes it possible to measure the breaking strength: paint/substrate. The average paint thickness is calculated from 10 measurements on each sample with the ELCOMETER 345 FNF S-MKI gauge according to ISO: 2409-1994.

The paint consists of three layers that are 200 µm thick.

The degree of roughness was measured by the total height of the formed roughness during surface preparation. The value of the full amplitude between the peak and valley, which is 25 to 50 µm, is acceptable.

#### 2.3.4. Chamber of the Salt Spray

**Figure 2:**
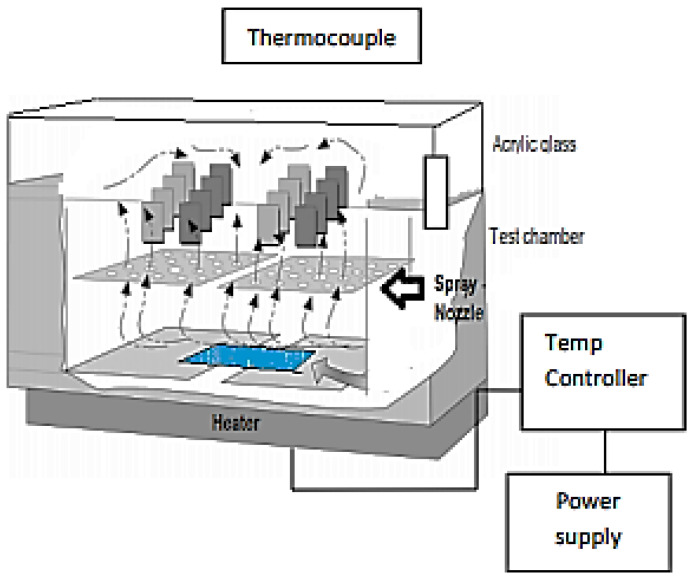
Block diagram of corrosion chamber.

This permits the realization of the resistance tests at the salt spray following the French norm AFNOR X41-002 [11]. It is entirely constituted by a test tank in armed polyester glass and unattackable to the salt spray. The outer body is in rigid steel sheet metal, lacquered gray and hammered. The cover in the shape of the roof is transparent; the Plexiglas permits the direct observation of the pieces in the test. It includes a thermometer to control the internal temperature and supports studying the pieces. The heating of the tank was ensured by an electric resistance surrounding it and controlled by a thermometer (Figure 2). The test simulates the atmospheric conditions near the sea while being based on the rules of actual correlation (1 h of the salt spray corresponds to 15 days inside the sea). The witness’s plates, were put to the test with the saline fog for 340 h.

### 2.4. Electrochemical Measurements

A saturated calomel reference electrode (SCE) and a platinum grid counter electrode were used in the Potentiostat/Galvanostat Model Solatron 1255B frequency response analyzer for electrochemical research. Polytetrafluoroethylene tape (PTFE) was used to insulate a cylindrical C38 steel rod, resulting in an A = 7.5 cm^2^ surface area exposed to the solution. Uncoated and coated CS samples were immersed in soil extract from different locations at 30 °C for corrosion testing. The applied potential was scanned at 0.5 Mv per second [30,31]. Recordings of both anodic and cathodic fields were made.

At open-circuit potential (OCP), a Solartron SI1287 electrochemical interface was used for electrochemical impedance spectroscopy (EIS) from 2 mHz to 100 kHz, with an amplitude of 10 mV. View 2 software was used to model the impedance data.

### 2.5. Control Tests

The degree of care is defined to evaluate the surface condition required for good paint adhesion. It is the most common way to check the surface condition after Sa2.5 sandblasting. This compares the surface in question with photos according to ISO 8501, making it possible to determine the required degree of care and characterizing the more or less thorough removal of impurities.

The salt spray test is a standardized evaluation of the corrosion resistance of metallic materials, whether with or without a temporary or permanent corrosion protection coating. However, the salt spray test is a widely used method of quality control layers. The conducting of these tests is described by various standards (ASTM B1171, EN ISO 92273). In all cases, the parts to be evaluated are placed in a test chamber where a salt solution is sprayed at a specific temperature. The test can run continuously from six to over a thousand hours. The most corrosion-resistant materials can be tested for more extended periods. The salt spray test will give different results than corrosion under normal conditions. Corrosion is indeed a complex phenomenon.

### 2.6. Theoretical Study

#### 2.6.1. Quantum Chemical Calculations 

The ORGS and Fe_18_ cluster structures were optimized using the DFT-B3LYB functional and the Turbomole package’s TZVP basis set [32]. The Conductor-like Screening Model for Real Solutions (COSMO-RS) was computed using the COSMOTherm program [32,33,34]. Quantum chemical methods are rather helpful in elucidating the relationship between electronic structure and reactivity. Thus, quantum chemical calculations have been widely used in corrosion inhibition studies. Chemical reactivity descriptors such as electronegativity, softness, chemical potential, chemical hardness, and the HOMO–LUMO energy gap were calculated.

From the ionization (*I*) and electron affinity (*A*) ground states, we can deduce the hardness (*η*) and chemical potential (*μ*) or electronegativity (*χ*) equations, respectively.

(1) $$\chi =-\mu =\frac{I+A}{2}$$

(2) $$\eta =\frac{I-A}{2}$$

(3) $$I=-E_{HOMO}$$

(4) $$A=-E_{LUMO\;}$$

$E_{HOMO}$ and $E_{LUMO}$ represent the energy of the *HOMO* and *LUMO* orbitals, respectively.

The electronegativity and hardness values determine an ion’s, atom’s, or molecule’s electrophilicity index (*ω*). [35]

(5) $$\omega =\frac{\chi^{2}}{2\eta }$$

The percentage of electrons transported from the ORGS molecule to the CS sample’s surface is computed as follows [34]:

(6) $$\Delta N_{max}=-\frac{\mu }{\eta }$$

#### 2.6.2. Polymer Blends

The polymer’s miscibility was investigated with the help of the Material Studio Blends module. The thermodynamics of different materials may be estimated directly from their chemical structures. A force field and molecule structures are all needed to run the mixture simulation [36].

The thermodynamic definition of miscibility in mixes of various materials may be given. It is possible to follow the Flory and Huggins theory, which is based on the calculation of free energy for mixing, which defines the state of a mixture. It describes the compounds as either soluble or insoluble [36,37]:

(7) $$\frac{\Delta G}{RT}=\frac{\emptyset_{A}}{n_{A}}\;ln\emptyset_{A}+\frac{\emptyset_{B}}{n_{B}}\;ln\emptyset_{B}+\chi \emptyset_{A}\emptyset_{B}$$

*n_A_* and *n_B_* are the mol numbers of the two components and their corresponding $\emptyset_{A}$ and $\emptyset_{B}$ volume fractions. The first two terms give the mixture’s combinatorial entropy, always negative [38]. Flory–Huggins’s (*χ*) parameter is defined as follows [39]:

(8) $$\chi =\frac{E_{mix}}{RT}$$

The mixing energy, *E_mix_*, is calculated by dividing the free energy of the mixture by the sum of the pure component energies (base (*b*) and screen (*s*)) [40,41].

(9) $$E_{mix}=\frac{1}{2}Z\left(E_{bs}+E_{sb}-E_{bb}-E_{ss}\right)$$

*E_bb_, E_ss_, E_sb_, E_bs_*: binding energies of two molecules *b* and *a*, where *b* refers to “base” and *s* to “screen”. *Z* denotes the coordinate number [40].

## 3. Results and Discussion

### 3.1. Adhesion Result

The results for the steel coating under the treatment conditions indicated that the primary achieved a 2B rating with a 2 min treatment at 300 W. The adhesion of the PL to the surface of the steel at 300 W deteriorated. The treatment at 300 W brought a 3B classification to the IL. For the same treatment time at a power of 300 W, the FL passed to 5B. Longer treatment times led to a deterioration of the adhesion of the final layer (Table 2). The adherence was not optimal at 300 W (PL and IL).

### 3.2. Investigation of the Corrosion Protection Efficiently

The CS and coating surface treatments were studied electrochemically and gravimetrically in 3% NaCl aqueous solutions to better understand the corrosion prevention mechanism.

#### 3.2.1. Evolution of Abandonment Potential as a Function of Immersion Time

**Figure 3:**
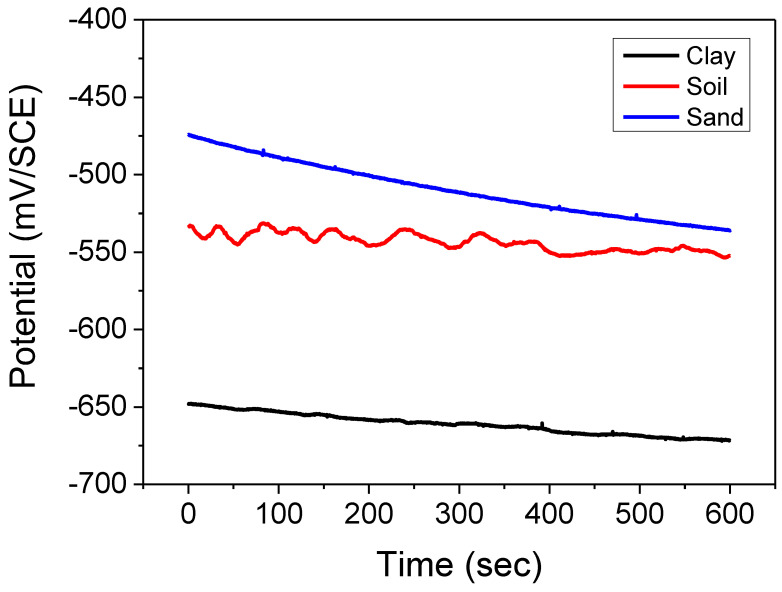
Potential abandonment vs. immersion time for the coated steel in the different soil extracts.

Monitoring the abandonment potential with the immersion time of the coated steel in a corrosive environment gives us information on the behavior of the metal surface concerning corrosion (dissolution or passivation). It enables us to determine the operating conditions of the steel according to its composition (addition of alloying elements) or the characteristics of the reagents (concentration) (Figure 3).

The shape of the curves (Figure 3) shows a decrease in the abandonment potential with the immersion time of the steel coated in the soil extracts (clay) and (Hamadi Krouma soil), reflecting a continuous degradation of the metal surface. We explain the negative evolution of the potential by an abrupt attack of the steel through the coating. For the soil extract (Bouzaaroura sand), we observed stability with fluctuations, reflecting the stable and noble values of the possible following permanent protection of the steel [41,42].

The values of the potentials and the curves were plotted to show that the soil extract’s initial abandonment potential is more electronegative than those obtained in the other two electrolytes, thus reflecting the activation of the surface (coating/metal). This can be explained by the reaction between the aggressive medium and the system (lining/metal [43]). 

The potential stability is linked to a dynamic equilibrium between the oxidation-reduction reactions.

#### 3.2.2. Potentiodynamic Polarization Curves

The values of corrosion current density (*I_corr_*), corrosion potential (*E_corr_*), cathodic and anodic Tafel slopes (*bc* and *ba*), and the inhibition efficiency *E* (%) for different soil extracts are given in Table 3 and represented in Figure 3. The inhibitory efficacy is defined as follows [44]:

(10) $$E=\left(\frac{i_{corr}-i\prime_{corr}}{i_{corr}}\right)*100\;$$

where $i\prime_{corr}$ and $i_{corr}$ are the steel corrosion current density values determined by extrapolation of the Tafel cathode lines after immersion in the various soil extracts.

**Figure 4:**
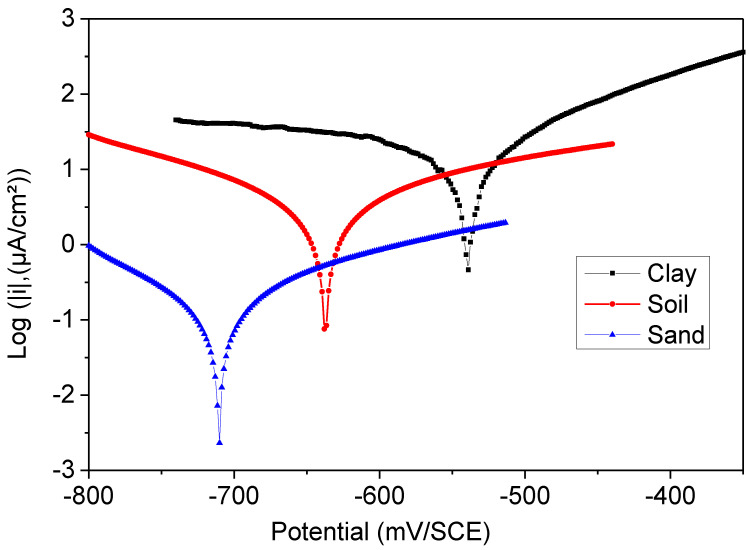
Polarization curve (Tafel) of the coated steel in the different soil extracts, clay, soil, and sand.

Figure 4 illustrates the potentiodynamic polarization curves for coated steel in various soil extracts. Even for the soil extract sample of Ali Mendjeli, the anodic partial current increased much higher than in the cathodic domain. Generally, the impact of electrolytic conductivity is mainly anodic. The anomaly current decreased significantly in the second area of high polarization potential for both soil extracts [25,31]. There was no influence on the anode curves. The low aggressivity of these extracts may be a factor in this. It dissolved more slowly in this situation.

Extraction of the soil sand extract reduced the corrosion current density (*i_corr_*) and coated steel’s corrosion potential (*E_corr_*). Sand soil *i_corr_* reduced from 97 μA/cm^2^ to 56 μA/cm^2^ for clay soil. *E_corr_* saw a negative (cathodic) change from −0.681 V vs. SCE to −0.650 V. The polymer film resistance of the coated steel rose from 82.5% for sand soil to 89.9% for clay soil.

#### 3.2.3. Electrochemical Impedance Spectroscopy 

**Figure 5:**
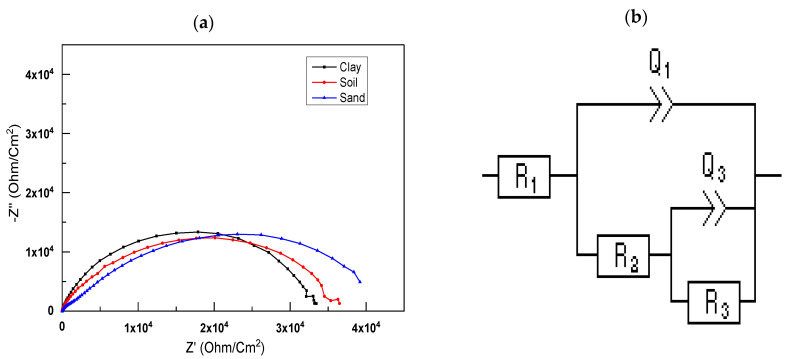
(**a**) Nyquist plots of the coated steel in the different soil extract, (**b**) Equivalent electric circuit.

The open-circuit potential amplitude of 10 mV was used to conduct further electrochemical impedance experiments, along with a frequency range of 2 mHz to 100 kHz. Figure 5a shows a Nyquist plot for different soil extracts. Coated steel has one significant material constant in the high- and medium-frequency bands, represented in Figure 5a.

Figure 5 shows the EIS Nyquist plots of substrates covered with different soil extracts generated using a basic equivalent electric circuit model Figure 5a,b. It includes factors such as RS (solution resistance), *R_p_* (polarization resistance), and *C_dl_* (coating capacitance) [45]. The constant phase element (Q3) teaches us about the behavior of polymer coatings. A breakdown of these metrics can be seen in Table 4.

Variations in the diameter of the soil extract capacitive loops are shown in Nyquist plots. The semicircles are not quite round. Those capacitive loops reflect substrate corrosion controlled by the charge transfer mechanism of corrosion. Semicircle diameters shrank for samples soaked in Hamadi Karouma and Ali Mendjeli soil extracts. The following figure illustrates this point: (a) the creation of capacitive loops may indicate substrate corrosion. The substrate’s capacitive circle diameter in the crosslinked sand soil extract solution was greater than the preceding two soils, which means superior corrosion resistance [46,47]—the greater the diameter of the capacitive loop, the more corrosion-resistant the sample solution is. The latter has a more considerable polarization resistance (*R_p_*) of 11.816 Ohm cm^2^, indicating the potential of a highly compact coating (without porosity) by the coating solution employed compared to the other samples and high capacitance *C_dl_* = 1.16 × 10^−5^.

### 3.3. Cathodic Delamination 

#### 3.3.1. Polarization Resistance as a Function of Immersion Time

**Figure 6:**
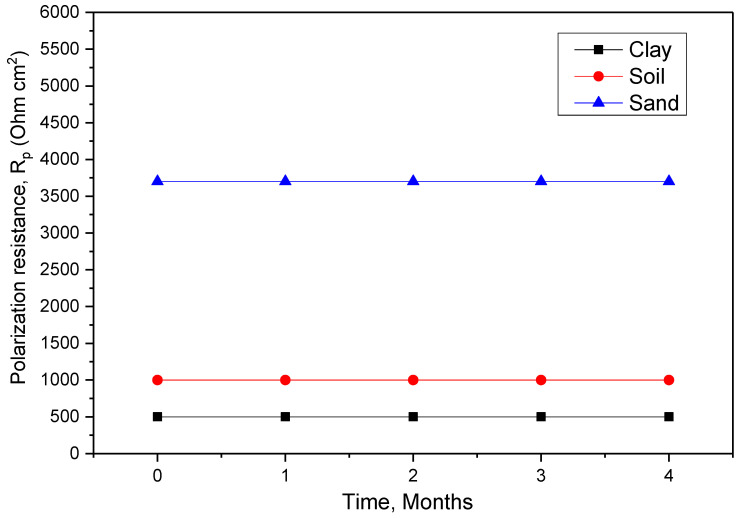
Polarization resistance as a function of immersion time of coated steel.

Corrosion rate measurements using the polarization resistance technique consisted of plotting the “intensity potential” curves of the coated steel in the study medium by sweeping the 10 mV/ECS potential in the vicinity of the equilibrium potential at a sweep rate of 100 mV/min corrosion for the coated samples in a different extract of soil and monitoring the corrosion current. The results obtained are shown in Figure 6 and grouped in Table 5. The overall evolution of the curves seems similar for the set of samples.

Indeed, for the coated sample (1 month of immersion), the polarization resistance *R_p_* showed a very high value and a low current density. This is due to the stable layer that adheres to the surface of the steel after 12 months of immersion. The *R_p_* value decreased for large current values as a function of time. This is due to infiltration through the coating of the electrolyte solution and degradation as the immersion time increases for all extract of soil.

These values are consistent and generally reflect the effect of immersion time on the degradation of the protective capacity of the system as a function of immersion.

#### 3.3.2. Cathodic Loading

*Cathodic Loading (Potentiostatic Polarization Curves)* 

**Figure 7:**
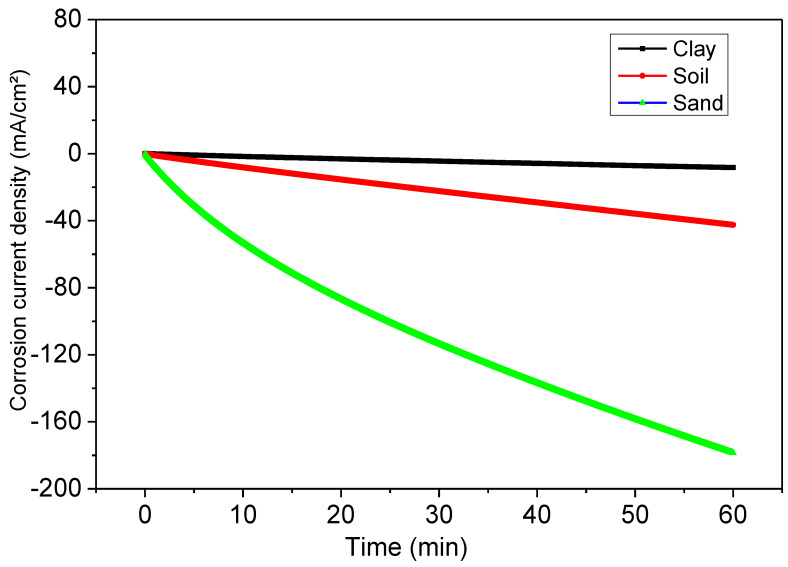
Potentiostatic polarization curve (cathodic loading) of coated steel for 1 h.

The method of bias curves in the potentiostatic regime (cathodic loading) follows the evolution of the current as a function of time for a constant imposed potential value (Figure 7). The tests were carried out at an imposed potential *E* = −1200 mV/ECS for immersion of 1 h.

The successively obtained curves for the system as a function of the immersion time showed a noticeable evolution of the current density from the cathode zones to the anode zones as a function of the immersion time. This remarkable evolution of the system could be linked to the infiltration of the solution following the degradation of the paint in the soil extract of Hamadi Krouma. The weak current change for the first immersion times reflects the excellent adherence of the stain to the substrate.

### 3.4. Observation of Surface Condition by SEM after Cathodic Loading

Visual examination of the metal surface of the coated steel (cathodic loading): There was a significant surface degradation. Dents showed the loss of adhesion of the coating layer. None of the bumps observed were open for the sample, but cracks appeared on the surfaces of the painted pieces in the extracted soil.

Accelerated aging methods are often indispensable tools for laboratory tests [9,48]. This property has been verified by comparing steel samples protected by salt spray aging coatings with samples subjected to prolonged immersion in extracted soil solution [9].

### 3.5. Study of the Aging of Organic Coatings by Salt Spray

The evolution of paint degradation on the control plates was evaluated by the rate (in %) of damage to the coated surface for the paint system.

**Figure 8:**
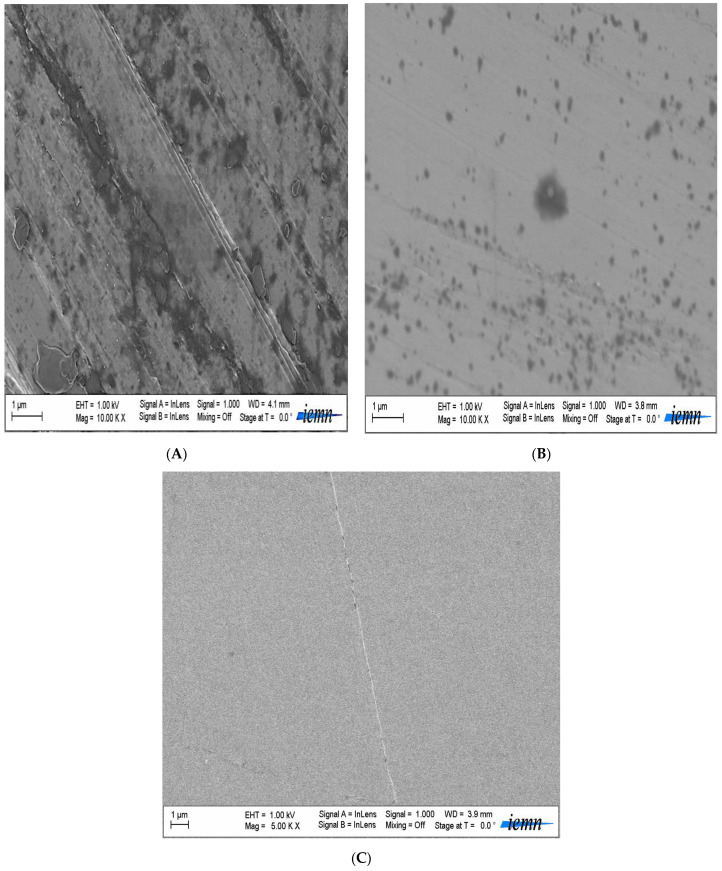
SEM images of the coated steel in the different soil extracts, (**A**) clay, (**B**) soil, and **(C**) sand.

The salt spray test pulverized the witness plates with the features following the French norm AFNOR X41-002 [19]. During 336 h, the saline solution was 5%, the inside temperature of the pulverization room was 35.8 °C, and the pressure was 0.1 MPA. The evolution of the paint’s degradation on the witness plates is represented in Figure 8, which was estimated by the rate in percent of the damaged coated surface. The rate of the damaged surface is in hours and a year of exposure to the salt spray (see the Supplementary Data), which is gathered in Table 6.

**Figure 9:**
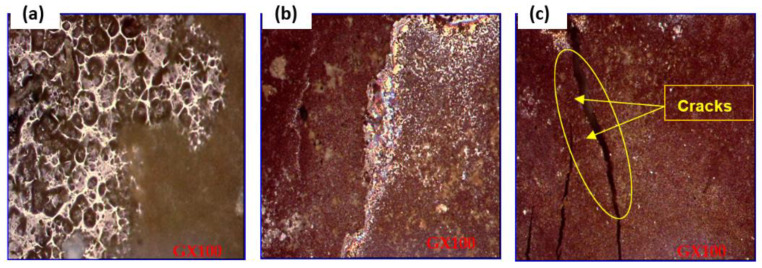
Metallographic observation of coated samples (cathodic loading) in the GX100 salt fog chamber of the coated steel in the different soil extracts, (**a**) clay, (**b**) soil, and (**c**) sand.

Around 162 h corresponding to 7 years of exposure to salt spray, the paint system remained intact (% damage after eight years equal to 0%). After eight years of exposure, the system showed surface attack (presence of corrosion points), and the paint damage rate was 10%. After 336 h, corresponding to 14 years of exposure, the paint was damaged by 40–55%. After 15 years and three months of exposure (366 h), the paint damage exceeded 58%, and significant corrosion was remarkable after 17 years, with a damage rate of 80%. These observations showed that blisters appeared during the first 15 h of exposure. They remained mostly closed after 40 h; SEM observation (Figure 9) showed that some blisters were cracked. Observation after 80 h showed that the coating was flaking. We can then consider that the damage to the layer takes place in several stages.

### 3.6. Theoretical Study

#### 3.6.1. DFT Study

The electrophilicity index (*ω*) is similar to chemical hardness and potential in global reactivity. This reactivity index indicates the presence of an additional charge (Δ*N*). While electrophiles may take electrons from their environment, their energy must drop. The molecule’s electronic chemical potential determines the charge transfer direction. As a result, its electrical and chemical potentials must be negative. Chemical reactions often include nucleophiles with a lower electrophilicity index between two molecules. In the investigated system, the EPAA operates as a nucleophile in the ECCR/EPAA contacting layers and as a nucleophile in the EPAA/PUAk contacting layers. The PUAk is a nucleophile, while the ECCR is an electrophile.

The electrophilic charge transfer (*ECT*) is defined as [46]:

(11) $$ECT={\left(-\frac{\mu }{\eta }\right)}_{A}-{\left(-\frac{\mu }{\eta }\right)}_{B}$$

For *ECT* > 0, charge flow from *B* to *A*; for *ECT* < 0, charge flow from *A* to *B*. The calculated *ECT* values (Table 7) confirm that electron flows from the ECCR to the EPAA (*ECT* < 0) and from the EPAA to the PUAk (*ECT* < 0).

**Figure 10:**
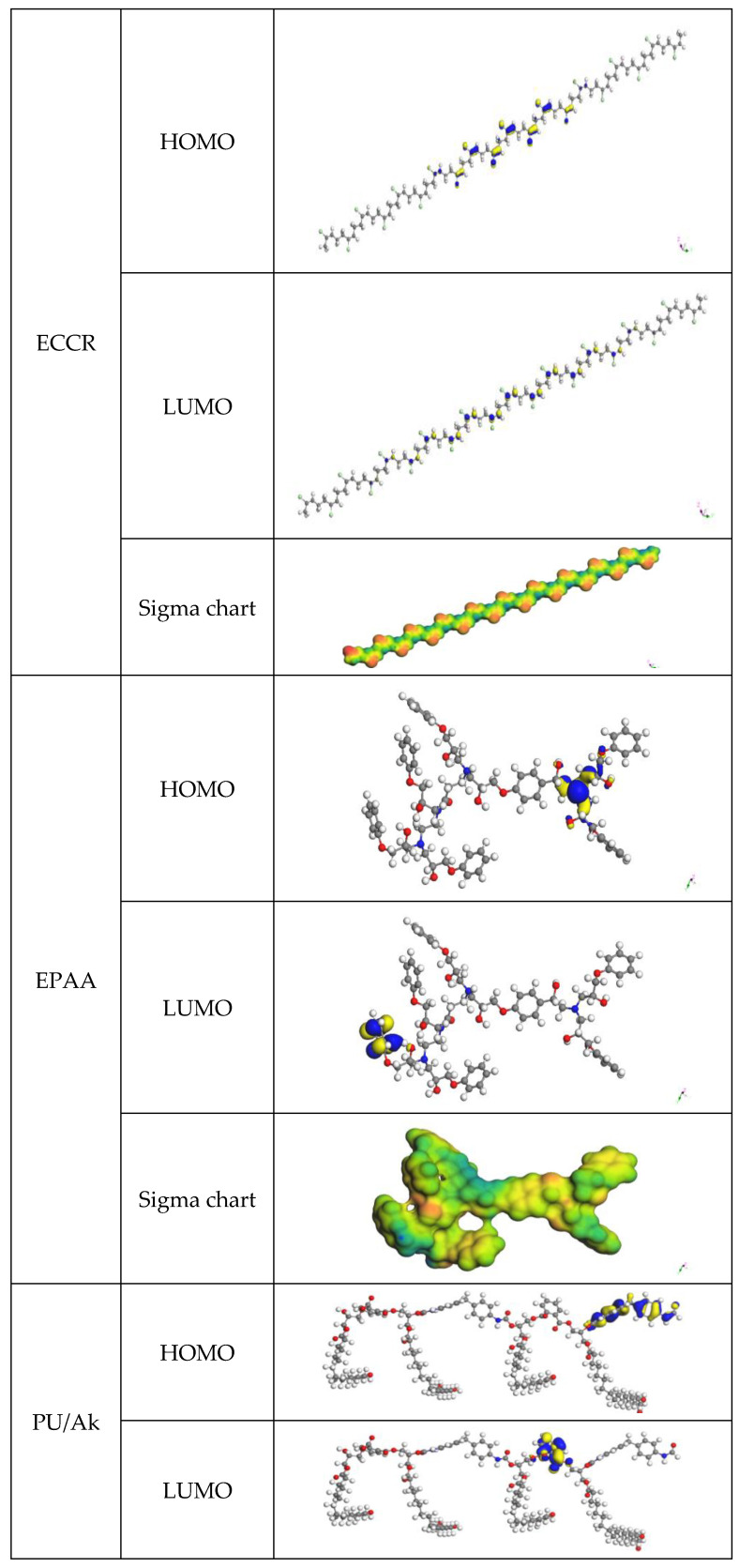

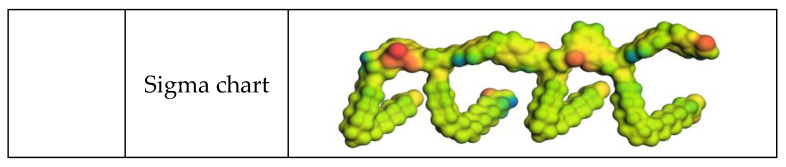
Three-dimensional plots of HOMO, LUMO, and σ-chart.

When these orbitals are intertwined, the frontier molecular orbitals (FMO) theory claims that the reaction’s rate may be accurately predicted. An electrophilic species would attack where the HOMO density is greater. Comparatively, there would be a greater concentration of LUMO in a region where a nucleophilic species would attack. The frontier orbital energy gap tells us about a molecule’s chemical reactivity and kinetic stability. The ability to polarize molecules with small orbital gaps is more noteworthy than other molecules. Some refer to this compound as “soft” because of its low kinetic stability and high chemical reaction rate. The HOMO orbital serves mainly as an electron donor. The LUMO orbital is the primary electron acceptor. The border orbitals and sigma chart are provided in Figure 10. More stability was found for the ECCR than for the PUAk, which was more reactive.

#### 3.6.2. Blends Study

The mix of the interacting systems, namely the ECCR, EPAA, and PUAk, is summarized in Table 8. The findings revealed that the EPAA (second layer) and the PUAk (third layer) have lower mixing energy (6.16 kcal mol^−1^ at 298.15K with χ = 10.39) than the ECCR (first layer) and the EPAA (second layer) (80.79 kcal mol^−1^ at 298.15K with χ = 136.43). The Flory–Huggins measure indicates that mixing the EPAA and PUAk is simpler than mixing the ECCR and EPAA. The estimated coordination number suggests that the distributions of the two polymers at the two interfaces (first/second; second/third) are equivalent. The system with eight molecules of the EPAA around the PUAk has a higher Z number. This enables deducing that the EPAA operates as a separation layer.

**Figure 11:**
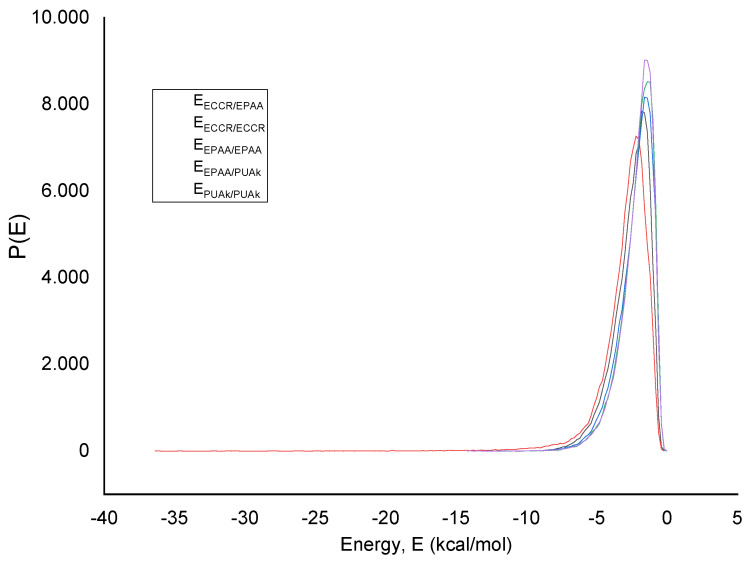
Mixing energy distribution plot.

The mixing energy distribution is seen in Figure 11. The generated Boltzmann weighted charts demonstrate unequivocally that the whole system is compatible with overlapping graphs.

## 4. Conclusions

CS was coated with three layers of polymers known for their remarkable corrosion resistance as a preventative measure.

Evidence from electrochemical investigations showed without a doubt that the polymer improved the corrosion resistance of steel coatings in soil extract over 60 days and under external polarization circumstances. A more excellent barrier creates a protective layer effect, which is why such a medium resists corrosion better than the others.

Blistering, spalling, and cracking are all damage that may be linked to specific physical occurrences in soil. Thanks to scanning electron microscopy, this is now much easier to determine.

The maximum (56 µAcm^−2^) average corrosion current density (*i_corr_*) of the total electrode surface area measured by PD occurred at an estimated efficiency of 89.9% for sand extract, and maximum (97 and 74 µAcm^−2^) aggressivity occurred between 82% and 86.6%.

The electrochemical impedance spectroscopy experiments showed that the impedance changed significantly with the immersion time. According to the results, there is always a capacitive and resistive interaction between the material and medium. The longer one is immersed, the less resistant one becomes. The aggressive species in the medium impregnated the protective layer formed by the porosity of the coatings, causing the process to occur. The results of the EIS polarization resistance also confirmed these findings.

## Figures and Tables

**Table 1:** Chemical composition of soil extracts and percentage.

Soil Sample	Percentage (%)			
	SO_4_^2−^	Cl^−^	CaCo_3_	M.O
Clay	Traces	0.23	2.81	1.74
Soil	Traces	0.24	56.80	0.20
Sand	Traces	0.21	3.80	0.10

**Table 2:** Adhesion result for the steel coating under the treatment conditions of 2 min at 300 W.

		PL	
processing power (W)	300	2B	0B
		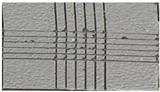	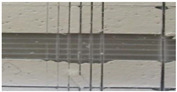
	IL		
	300	2B	3B
		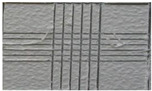	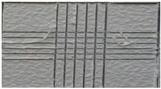
	FL		
	300	4B	5B
		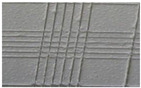	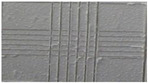

**Table 3:** Electrochemical parameters and corrosion-inhibiting efficiency of the coated steel.

Samples	*E_corr_* vs. SCE (mV)	*I_corr_* (μA cm^−2^)	*b_a_*_1_ (mV dec^−1^)	*b_c_* (mV dec^−1^)	*E* (%)
clay	−681	97	80	166	82.5
soil	−575	74	79	165	86.6
sand	−650	56	76	163	89.9

**Table 4:** Impedance parameters and corrosion resistance of the coated steel.

	R_1_ (Ohm)	*C_dl_* (F. s^n−1^)	R_ct_ (Ohm)	Q_3_ (F. s^n−1^)	*R_p_* (Ohm)
Clay	3.23	1.35 × 10^−5^	0.2436	7.60 × 10^−6^	38,451
Soil	2.874	9.22 × 10^−6^	3246	3.85 × 10^−5^	39,946
Sand	3.266	1.16 × 10^−5^	11,816	8.81 × 10^−6^	21,386

**Table 5:** Polarization resistance corrosion current and corrosion potential (for clay).

Immersion Time (Months)	1	3	6	12
*Rp* (W·cm^2^)	3200	1500	900	500
*E* (mV/ECS)	−405	−458	−527	−567
*I* (mA/cm^2^)	0.12	0.37	0.47	0.69

**Table 6:** Rate of surface damage as a function of the number of hours of exposure to salt spray (clay).

Temps (h)	3	18	24	35	48	66	72	96
Substrate	Intact	Intact	Intact	Intact	Intact	Intact	Intact	Intact
Time (h)	138	144	162	186	192	210	216	226
Substrate	Intact	Intact	Intact	Intact	Intact	Intact	Intact	Intact
Time (h)	232	238	258	264	282	288	306	330
Substrate	Intact	Intact	Start of attack	Superficial attack 2%	Superficial attack 4%	Superficial attack 10%	Superficial attack 18%	Superficial attack 20%
Time	336	342	360	366	384	390	404	420
Substrate	Attack 24%	Attack 28%	Attack 30%	Attack 50%	Attack 56%	Attack 60%	Attack 66–75%	Attack 80%

**Table 7:** Global reactivity descriptors.

	HOMO (eV)	LUMO (eV)	GAP (ev)	*χ*	*η*	*ω*	Δ*N_max_*	*ECT*
ECCR	−5.423	−1.242	4.181	3.332	2.091	2.656	1.594	
								−0.874
EPAA	−4.648	−1.967	2.681	3.308	1.340	4.081	2.468	
								−0.573
PUAk	−5.189	−2.621	2.568	3.905	1.284	5.938	3.041	


**Table 8:** Blend of the interacting systems: energies are in kcal mol^−1^.

Base	Screen	*χ* (298 K)	*E_mix_* (298 K)	*Z_bb_*	*Z_bs_*	*Z_sb_*	*Z_ss_*
ECCR	EPAA	136.43	80.79	5.36	5.90	4.70	5.59
ECCR	PUAk	90.14	53.38	5.36	5.08	5.88	5.49
EPAA	PUAk	10.39	6.16	5.59	4.15	7.23	5.49

## Data Availability

The data presented in this study are available on request from the corresponding author.

## Supplementary Materials

The following supporting information can be downloaded at: https://www.mdpi.com/2073-4360/14/16/3288/s1, Table S1: Characteristics of the intermediate and top coat.
