# Supplementary material for: Electrochemical and Computational Approaches of Polymer Coating on Carbon Steel X52 in Different Soil Extracts
Source: Polymers (Basel). 2022 Aug 12;14(16):3288. doi: 10.3390/polym14163288 (PMC9460235; doi:10.3390/polym14163288)
Supplement: Supplementary file 1 [file polymers-14-03288-s001.zip › polymers-1812498-supplementary.pdf]

Electrochemical and Computational Approaches of Polymer Coating on Carbon Steel X52 in Different Soil Extracts

Hana Ferkous [1,2], Amel Delimi [1,2], Abdesalem Kahlouche [3], Chérifa Boulechfar [1,2], Souad Djellali [4], Amina Belakhdar [5], Krishna Kumar Yadav [6], Ismat H. Ali [7], Akil Ahmad [8], Hyun-Jo Ahn [9], Magda H. Abdellattif [10], Byong-Hun Jeon [9,*] and Yacine Benguerba [11,*]

[1] Laboratoire de Génie Mécanique et Matériaux, Faculté de Technologie, Université de 20 Août 1955 de Skikda, Skikda 21000, Algeria; hanaferkous@gmail.com (H.F.); a_delimi03@yahoo.fr (A.D.); boulechfarcherifa54@gmail.com (C.B.)

[2] Département de Technologie, Université de 20 Août 1955 de Skikda, Skikda 21000, Algeria

[3] CRTI Research Centre in Industrial Technologies-CRTI, P.O. Box 64, Cheraga, Algiers 16014, Algeria; ka_salem@yahoo.fr

[4] Laboratoire de Physico-Chimie des Hauts Polymères (LPCHP), Faculty of Technology, University Ferhat Abbas Setif1, Setif 19000, Algeria; djellali.souad2006@gmail.com

[5] Laboratoire Matériaux et Systèmes Electroniques, Université de Bordj Bou Arreridj, Bordj Bou Arreridj 34000, Algeria; amina_bel@hotmail.fr

[6] Faculty of Science and Technology, Madhyanchal Professional University, Ratibad, Bhopal 462044, India; envirokrishna@gmail.com

[7] Department of Chemistry, College of Science, King Khalid University, P.O. Box 9004, Abha 61413, Saudi Arabia; ihali@kku.edu.sa

[8] Chemistry Department, College of Science and Humanities, Prince Sattam Bin Abdulaziz University, Al-Kharj 11942, Saudi Arabia; aj.ahmad@psau.edu.sa

[9] Department of Earth Resources and Environmental Engineering, Hanyang University, Seoul 04763, Korea; hjahn93@hanyang.ac.kr

[10] Department of Chemistry, College of Science, Taif University, Al-Haweiah, Taif 21944, Saudi Arabia; m.hasan@tu.edu.sa

[11] Laboratoire de Biopharmacie et Pharmacotechnie (LBPT), Ferhat Abbas Setif 1 University, Setif 19000, Algeria

* Correspondence: bhjeon@hanyang.ac.kr (B.-H.J.); yacinebenguerba@univ-setif.dz (Y.B.)

Supplementary data: Table S1

Characteristics of the intermediate and top coat			
	PL	IL	FL
Nature	Epoxy Chloric cc marine PZ	Epoxy/polyaminoamide	Polyurethane /alkyde
Utilization	Zinc-rich anticorrosion Under Coat<br>For steel structures in marine and marine and industrial environments	For steel structures in marine	Two-component finish for industrial epoxy system
Proprieties	Good adhesion<br>Good corrosion resistance<br>Fast drying	Good adhesion<br>Reinforces the corrosion resistance<br>Corrosion resistance of the system	Good outfit performance<br>Good adhesion<br>Good resistance to environmental conditions
Characteristics	Density:1,422±0,05<br>Viscosity at 25°C: 1240 p0 ±50<br>Yield :3,5 m2/Kg or 11m2/l<br>Drying at 25°C: HT 20-30' sec 8-10h<br>EA/EB: 97/3<br>Pot time: 7-8h at 25°c<br>Aspect: matt	Density: 1.864<br>Viscosity at 20-25°C: 580 P0 ±30<br>Yield: 6m2/Kg<br>Drying at 20-25°C: HT 3- 4h<br>Storage time: 6 months dry<br>EA/EB: 90/10<br>Pot time: 7-8h<br>Aspect: Mat	Density: 0.98<br>Viscosity at 20°C: 80 ± 10 s<br>Drying time:<br>HT: 8 hours, Dry: 16 hours<br>Hard: 24 hours<br>Color: grey<br>Aspect: glossy<br>Mixing ratio by weight:<br>EA/EB: 80/20<br>Pot life: 7 to 8 hours at 20°C<br>Stamping (7mm): Good<br>Folding (8mm): good<br>Shelf life under cover: 1 year
